# Morphological changes of the pancreas after pancreaticoduodenectomy

**DOI:** 10.1038/s41598-019-51173-1

**Published:** 2019-10-10

**Authors:** Rita Quesada, Clara Simón, Aleksandar Radosevic, Ignasi Poves, Luis Grande, Fernando Burdío

**Affiliations:** 1 Experimental and Health Sciences Department, Universitat Pompeu Fabra, Barcelona, Spain; 2 School of Medicine, Universitat Pompeu Fabra and UAB, Barcelona, Spain; 3 Radiology Department, Hospital del Mar, Barcelona, Spain; 4 General Surgery Department, Hospital del Mar, Barcelona, Spain

**Keywords:** Pancreatic cancer, Computed tomography, Outcomes research

## Abstract

The aim of this retrospective study was thus to evaluate postoperative morphological changes in the remnant pancreas after pancreaticoduodenectomy (PD) associated with postoperative pancreatic fistula (POPF). Fifty-one patients subjected to PD were enrolled in the study and allocated into 2 groups according to the presence (n = 16) or absence of POPF (n = 35). A morphological evaluation of the pancreas was conducted for up to a 20 months follow-up on CT scans and compared between groups. No significant differences were observed in morphology between the groups at the different preoperative and PO intervals, regardless of the clinical relevance of the POPF or POPF grade. However, in the overall patient analysis we observed a significant reduction of the entire pancreas over time. In fact, thickness decreased 0.4 mm/month, length 1.2 mm/month and volume 1.17 cm^3^/month over the PO. The impact of age, POPF, type of anastomosis, surgical technique and PO follow-up (time) was evaluated in a multivariate analysis using the general linear model, but only PO follow-up had a significant influence on the final model (p < 0.001). A significant reduction on pancreatic parenchyma (thickness, length and volume) occurs after PD with no significant differences between patients with or without POPF.

## Introduction

Pancreaticoduodenectomy (PD) with or without pylorus preservation has emerged as a standard therapeutic method for treating either malignant or benign diseases of the pancreatic head and/or periampullary region^1^. Although, recent advances in perioperative management have helped to reduce the mortality rate associated with PD to below 5% in high-volume centers, morbidity remains high, ranging from 30% to 50%^2^. Of all the possible complications, postoperative pancreatic fistula (POPF) has traditionally been regarded as the most frequent major complication and is a potentially serious, life-threatening event that may also cause delayed gastric emptying, prolong hospital stay and increase costs^2–4^. Despite all the advances and technical modifications in POPF prevention during this past decade, the incidence of this dreaded complication still ranges between 3–45% even in experienced hands^3,5,6^. The International Study Group of Pancreatic Fistula (ISGPF) recently redefined clinically relevant POPF as a drain output of any measurable volume of fluid with an amylase level >3 times the upper limit of institutional normal serum amylase activity, associated with a clinically relevant development or condition related directly to the postoperative pancreatic fistula^7^. Some of the recognized POPF risk factors are excessive blood loss, main pancreatic duct size <3–5 mm, soft pancreatic parenchyma, BMI and certain disease pathologies^8^.

Following PD, gastrointestinal pancreatic drainage can be restored by a number of different techniques. No studies have revealed significant differences in the rate of complications using different pancreatic anastomosis techniques^9,10^, although some have observed the frequent impairment of the pancreatic juice outflow due to stenosis of the pancreaticoenterostomy and atrophy of the pancreas associated with pancreatic exocrine insufficiency^9,11–13^. According to Sabater *et al*.^14^, PD is associated with pancreatic exocrine insufficiency in 58.6% of cases, which is comparable to those reported by Matsumoto *et al*.^13^ and Fang *et al*.^15^ with values of 50% and 52.4%, respectively^13,15^. When comparing the function of the remnant pancreas, morphological evaluation may be useful, as the pancreatic function depends on the volume of the pancreatic parenchymal tissue. In fact, pancreatic enzyme supplementation should be considered preoperatively in many patients and in almost all cases postoperatively because of the parenchymal reduction associated with PD^13^. The most likely explanation of pancreatic atrophy may not only be preoperative factors, such as preexisting obstructive pancreatitis, but also postoperative factors such as quantitative diminution of the pancreatic parenchyma as a result of the resection, stenosis of the pancreaticoenterostomy, malnutrition and deregulation of pancreatic neurohormonal stimulator mechanisms.

Complications such as POPF could in fact have an important effect on the parenchymal atrophy of the remnant pancreas due to chronic inflammation, obstruction or stenosis of the anastomosis^16^. However, very little information is still available on the morphological changes after PD, since the relationship between the morphological changes of the pancreas and POPF has not yet been evaluated. The aim of the present study was thus to evaluate the possibility of postoperative morphological changes in the remnant pancreas after PD in patients with and without POPF.

## Results

### Clinical findings

Table 1 shows the baseline characteristics of the patients included in the analysis. According to the above significance threshold, no differences were found in variables between the groups, except in the mean age, which was higher in the POPF group (p = 0.032). Concerning the PO characteristics, the hospital stay seems to be longer in the POPF group, and the percentage of patients who received enteric-coated pancreatic enzymes was also similar between groups, not being significant different in any case. Concerning the endocrine function, the ratio of preoperative diabetes was similar between groups and just four patients had new onset postoperative diabetes (3 in non-POPF group and 1 in POPF group), neither being significant in any case.

**Table 1:** Baseline characteristics of the patients involved in the study.

	Non-POPF group (n = 35)	POPF group (n = 16)	Total	p
Sex (male/female)	18/17	11/5	29/22	0.246
Age (years)	62.1 ± 13.4	70.4 ± 10.2	64.7 ± 12.9	0.032
**Consistency of the pancreas**				
Soft/Normal	14 (40.0 %)	10 (62.5%)	24 (48%)	0.291
Fibrotic	20 (57.1%)	6 (37.5%)	26 (52%)	
Unknow	1 (2.9%)	0 (0%)	1 (2%)	
**Type of tumour**				
Adenocarcinoma	23 (65.7%)	11 (68.7%)	34 (66.7%)	0.620
Cholangiocarcinoma	2 (5.7%)	0 (0%)	2 (3.9%)	
Others	10 (28.6%)	5 (31.3%)	15 (29.4%)	
**Type of anastomosis**				
PJ	26 (74.3%)	12 (75%)	38 (74.6%)	0.072
PG	8 (22.9%)	1 (6.3%)	9 (17.6%)	
None	1 (2.8%)	3 (18.7%)	4 (7.8%)	
**Surgical technique**				
PD	8 (22.9%)	8 (50%)	16 (31.3%)	0.530
PPPD	27 (77.1%)	8 (50%)	35 (68.7%)	
Duration of the intervention (min)	391 ± 79.9	342 ± 25.3	379 ± 72.7	0.184
Hospital stay (days)	16.6 ± 15.5	24.7 ± 14.8	19.6 ± 15.6	0.087
**ASA**				
I	3 (8.6%)	0 (0%)	3 (5.9%)	0.281
II	19 (54.3%)	7 (43.8%)	26 (51.0%)	
III	13 (37.1%)	9 (56.2%)	22 (43.1%)	
**Type of surgical approach**				
Open	24 (68.5%)	12 (75%)	36 (70.6%)	0.847
Laparoscopic	7 (20%)	2 (12.4%)	9 (17.7%)	
Conversion	3 (8.6%)	1 (6.3%)	4 (7.8%)	
Hand-assisted	1 (2.9%)	1 (6.3%)	2 (3.9%)	
Supplementary enzymes	20 (57.1%)	9 (56.3%)	29 (56.9%)	0.952
Preoperative diabetes	10 (%)	9 (56.3%)	19 (37.2%)	0.058
New onset postoperative diabetes	3 (8.6%)	1 (6.3%)	4 (7.8%)	0.775
Neoadjuvant chemotherapy	5 (14.3%)	1 (6.3%)	6 (11.7%)	0.409
Adjuvant chemotherapy	18 (51.4%)	8 (42.1%)	26 (50.9%)	0.925

PJ = Pancreatojejunostomy.

PG = Pancreatograstrostomy.

Pancreaticoduodenectomy (PD).

Overall, taking the above cited definition of POPF into account, 31.4% of the patients were diagnosed with POPF: 3 with Grade A (18.7%), 9 with Grade B (56.3%) and 4 with Grade C (25%). Four patients with POPF Grade B or C had other severe complications. The MPD was found to be dilated in 7 patients (18.8%) in the POPF and in 4 patients in the Non-POPF group (11.4%), the difference not being significant.

### Morphological analysis

**Figure 1:**
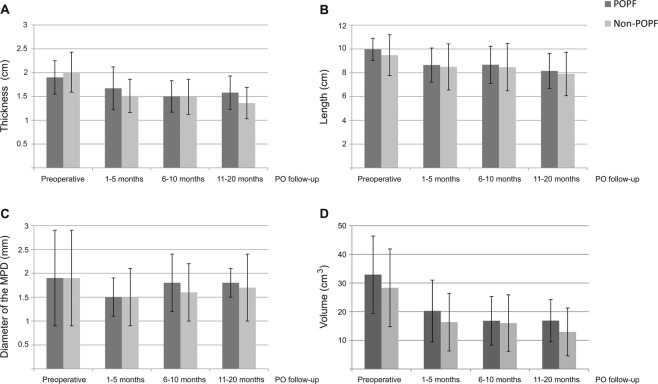
Mean thickness (**A**), length (**B**) and volume (**D**) of the pancreatic parenchyma and mean diameter of the main pancreatic duct (**C**) per group over the PO. MPD = Main pancreatic duct. No differences were observed between groups considering the presence of absence of POPF.

As shown in Fig. 1, no significant differences were observed in thickness, length, diameter of the MPD and volume between groups at the different preoperative and PO intervals, regardless of whether or not the POPF was clinically relevant or POPF grade.

**Figure 2:**
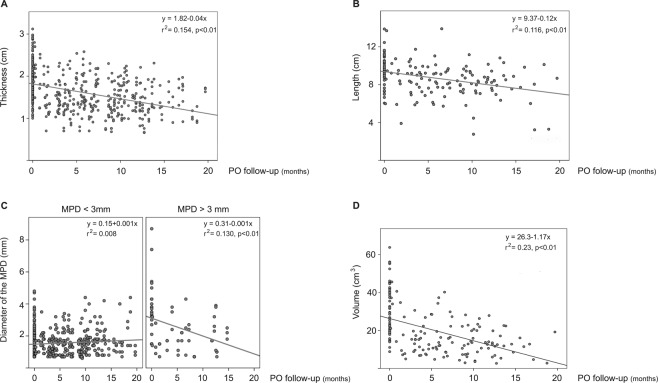
Scatter of all measurements of the pancreatic gland over the PO (n = 612), where (**A**) is thickness, (**B**) length and (**D**) volume of the pancreatic parenchyma. (**C**) Mean diameter of the main pancreatic duct for patients with MPD normal-size (<3 mm) and dilated (>3 mm). Best- fit equation for linear regression is represented by a line with the r^2^ value. All of the equations were statistically significant (p < 0.05) with the exception of normal-size MPD, but at the limit.

When we analyzed the morphological change in thickness, length, diameter of the MPD and volume over the PO without differentiating between groups, interesting changes were observed. The tendency for the thickness, length and volume of the pancreatic parenchyma to decrease over time was statistically significant (see Fig. 2). In fact, the best-fit equation for the linear regression analysis of the thickness indicated that the thickness of the parenchyma decreased 0.4 mm per month after the PD (r^2^ = 0.154; p < 0.01). This tendency was also observed in the length of the parenchyma, which decreased by 1.2 mm per month over the PO (r^2^ = 0.116; p < 0.001: Fig. 2B) and for volume, which decreased 1.17 cm^3^ per month over the PO (r^2^ = 0.23; p < 0.001: Fig. 2D).

No differences were observed in the mean diameter of the MPD over the PO in a global analysis. However, when we analyzed the mean MPD diameter in patients with previous dilation, we observed a significant decrease over the PO (p = 0.003). However, MPD diameter seemed to increase in the 44 patients who had a normal-size MPD, but at the limit of statistical significance (p = 0.062) (see Fig. 2C).

**Figure 3:**
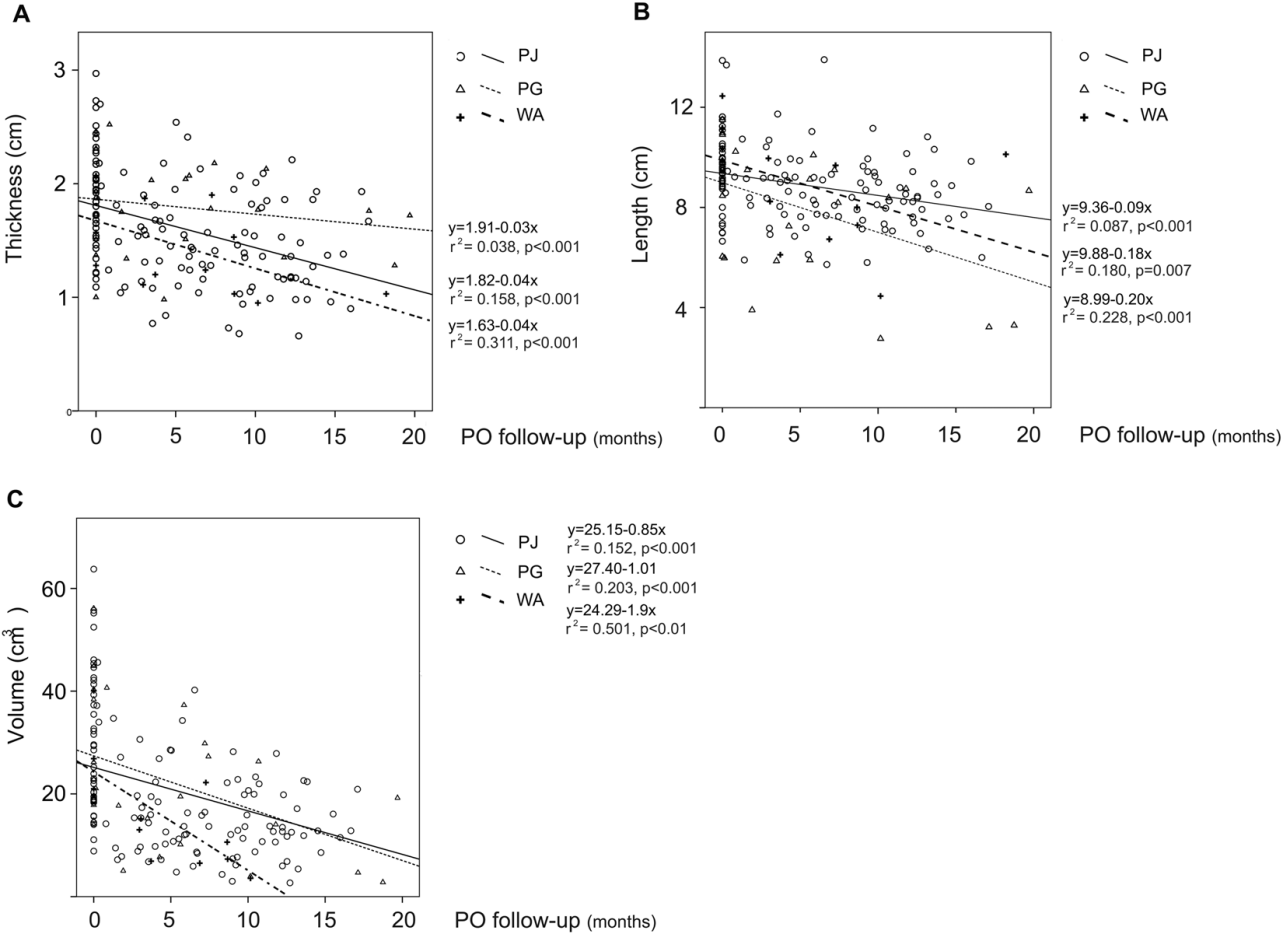
Scatter of all the mean thickness (**A**), length (**B**) and volume (**C**) of the pancreatic gland per patient and type of anastomosis over the PO. A minor decrease on length was observed for PJ than in PG or SA, while thickness showed a minor decrease in PG. Volume decreased considerably over the PO in patients without anastomosis. Best- fit equation for linear regression is represented by a line with the r^2^ value and the p value for each type of anastomosis.

When we analyzed the change in thickness and length over the PO differentiating between type of anastomosis, we observed a smaller reduction in thickness over PO for PG compared to PJ or WA (See Fig. 3A). While for length we observed a smaller decrease over PO for PJ than PG or SA (see Fig. 3A). In fact, the best-fit equation for the linear regression analysis indicated that the thickness of the parenchyma in patients subjected to PG decreased by just 0.3 mm per month. The decrease in length was 0.9 mm per month for PJ, and almost twice this figure for the PG and WA groups (2 mm/month and 1.8 mm/month, respectively). This could be explained by the telescoped PG performed, in which the cranial extremity of the remaining pancreas naturally protrudes into the gastric lumen^17^, so that a bigger decrease was found in length with time, but not in thickness. When we analyzed this by volume, we observed that both PG and PJ decreased similarly, while in the cases with no anastomosis the volume loss was greater, but not significantly so. As shown in Fig. 3C, the WA patients lost almost double the parenchymal volume (1.9 cm^3^/month) of those subjected to PJ or PG.

### Multivariate analysis of prognostic factors on the POPF

Seven variables were considered in the multivariate analysis based on the generalized linear model to evaluate the prognostic factors associated with the morphological changes of the pancreas: age, POPF, type of anastomosis, surgical technique, PO follow-up, preoperative diabetes and chemotherapy. However, only PO follow-up was found to have a significant influence on the final model, taking into account the same significance threshold (p < 0.01) for length, thickness and volume, while type of anastomosis (p < 0.05) also seems to influence changes in length, but not the other morphological variables.

## Discussion

Although technological improvements have significantly reduced the mortality associated with pancreaticoduodenectomy (PD) the incidence of postoperative morbidity remains high, with POPF the most significant cause of morbidity, at rates which vary according to the definition used^2–4^. A recent prospective multicenter randomized trial found POPF to be around 42%, regardless of the type of pancreatic anastomosis, in a subgroup of patients with soft pancreas and MPD < 3 mm diameter^18^. In our study we found a POPF ratio of 31.4%, similar to previous studies. When comparing the POPF group and the non-POPF group, no differences were found between the groups’ baseline characteristics, except in the mean age, which was higher in the former group. Advanced age (>70) has been widely described as a risk factor that influences POPF, as well as sex, operative time, texture of the remnant pancreas and pancreatic duct size, among others^19^. However, when age was considered in the multivariate analysis no significant influence of this factor was found in the final model.

Some authors have also suggested that pancreatic thickness, estimated pancreatic remnant volume and visceral fat on preoperative CT images can predict clinically relevant POPF. While these factors can in fact be objectively assessed, they are not as good predictors as the well-known risk factors cited above^20–23^.

POPF is evidence of the failure of the healing/sealing of the pancreatic-enteric anastomosis, or may be due to a parenchymal leak not directly related to anastomosis, such as one originating from the raw pancreatic-surface by any trauma2 or even by pancreatic manipulation. The pancreatic juice is rich in activated pancreatic enzymes capable of digesting adjacent tissues, thus generating chronic inflammation, which has been associated with obstruction or stenosis of the anastomosis site and therefore can cause pancreatic atrophy9,16, which could further compromise exocrine function^13,19^. However, in this study, after evaluating the morphological changes of the pancreas in patients with and without POPF through a general linear model, no association was found between the degree of parenchymal atrophy and the presence of POPF in patients that had undergone PD.

We also found similar rates of parenchymal reduction between both groups, with a mean reduction of 0.4 mm/month in thickness, 1.2 mm/month in length and 1.17 cm^3^/month in volume after 20 month of follow-up (See Figs 2 and 3). These results are aligned with those of previous studies^9,16,19,24^, which found a significant involution of the pancreatic parenchyma after PD, although the analyses were performed in different PO periods.

Concerning the type of anastomosis, many authors have compared PJ and PG with regard to the incidence of POPF, although no wide consensus has been achieved^16,25–27^. In this line, some studies^9,24,28^ have also analyzed the parenchymal changes on thickness after PD, and no significant differences were reported between PJ or PG. Only Tomimaru *et al*.^16^ have reported a significantly more reduced thickness in PG than PJ, which contrasts with our analysis, in which PJ seems to be associated with a higher loss of thickness than PG. If we focus on length, a variable which has not been studied in any of the previously cited studies, it seems to be lower in PJ than in the PG or WA group. Interestingly, these differences were not observed when we compared volume in the PJ and PG techniques, possibly because volume compensates for the differences between length and thickness. However, the results suggest that the parenchymal volume of patients subjected to PD without anastomosis (WA) seems to be related to severe atrophy.

Concerning the MPD, few studies have measured the diameter after PD, and those found no significant differences in the rate of change of the MPD diameter between PJ and PG^15,29^. However, Tomimaru *et al*.^16^ found not only significantly more severe atrophy of the pancreatic parenchyma in PG than PJ, but also changes in the MPD. They observed that a normal size MPD tended to become dilated before surgery in the PJ group. The current study also found a temporary change in MPD diameter related to dilatation of the MPD before surgery, though no differences were observed over the PO in a global analysis. Our results are consistent with those of the above-cited studies^9,16^. MPD diameter significantly decreased over the PO in patients with MPD > 3 mm, while it seemed to increase in the 44 patients who had a normal-sized MPD, but at the limit of statistical significance. This could be explained as a normal consequence when resolving passive obstruction of the duct due to neoplasia.

Few studies have been published to date on this issue and it has been suggested said that postoperative MPD dilatation and parenchymal atrophy may result from obstruction or stenosis of the anastomosis^9,30^. However, few studies have evaluated the permeability of the anastomosis after PD^31,32^.

As has been widely described^9,11,16,27,33^, atrophy of the pancreatic parenchyma frequently occurs after PD and is often associated with clinical and subclinical pancreatic exocrine, leading to a requirement for pancreatic enzyme supplements. However, in this study no evaluation was made of postoperative pancreatic exocrine or endocrine function.

Several studies have analyzed the morphological changes (pancreatic atrophy, duct dilation) after PD. However, one of the strengths of this study is that the role of POPF in the development of pancreatic atrophy has not been modeled up to now with consecutive images in time and space and a double methodology (based on scan images and on patients). On the other hand, the results obtained in the simple linear regression analysis are relevant at a clinical level, since inferences and predictions can be made about pancreatic thickness and length in PD patients.

Certain limitations of this study should be pointed out: firstly, as mentioned above, the functioning of the pancreatic remnant was not determined by any reliable test but only from clinical data (relief of digestive symptoms by oral enzymes), which shows the need for studies evaluating the effects of POPF and pancreatic atrophy on pancreatic function. Secondly, the number of POPF patients was relatively low, therefore its impact on the morphological changes in the parenchymal could be quite low and not statistically significant. It should also be noted that other factors such as a distal stomach resection could influence atrophy, as the stomach and duodenum are sources of atrophic stimulants such as gastrin or cholecystokinin.

In conclusion, our findings suggest that changes on the remnant pancreas after PD do not correlate with the occurrence of postoperative pancreatic fistula and that there is often a significant reduction in the thickness, length and volume of the pancreatic parenchyma.

## Methods

### Selection of patients

**Figure 4:**
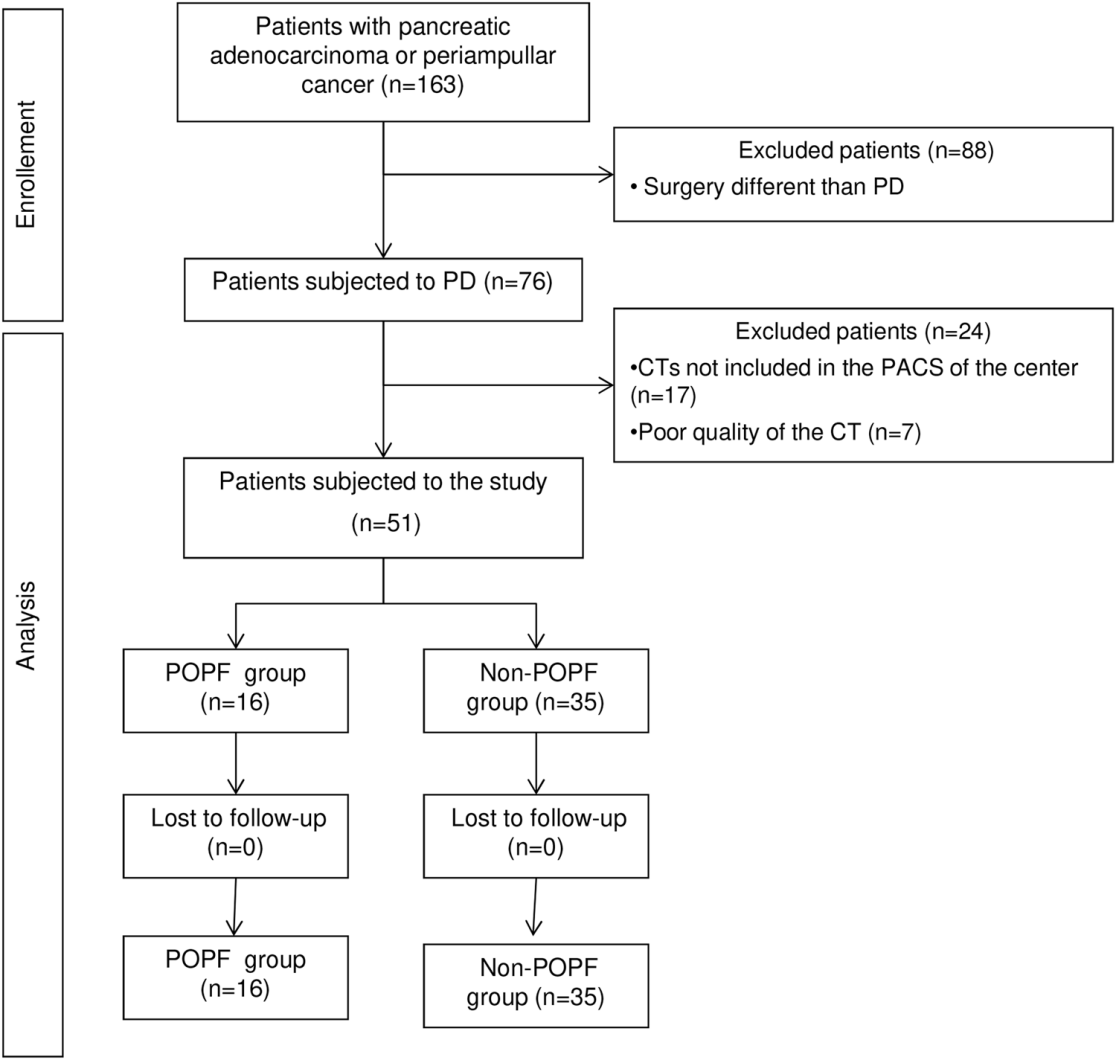
Flow chart of the study

A study population of 163 patients who had undergone pancreatic resection in the *Hospital del Mar* (Barcelona, Spain) between 2007 until 2013 was retrospectively analyzed and entered prospectively into a computer database. Informed consent was obtained from all participants and the protocol of the study was approved by the local ethical committee of the Parc de Salut Mar with reference number 2015/6468/I (CEIm-PSMAR) according with European Directive for Clinical Trials (Dir 93/42/CEE). The inclusion criteria were pancreatic adenocarcinoma or periampullar neoplasia. From the total number of patients, 75 had been subjected to standard PD. During the morphological analysis of the images, twenty-four patients were excluded because of absence or poor quality of the computer tomography (CT) which could negatively affect the measurements. With these criteria, 51 patients were enrolled in the study and were allocated into two groups according to the presence or absence of POPF: “POPF group” (n = 16) and “non-POPF group” (n = 35) (see Fig. 4).

### Morphological assessment of the residual pancreas

Examination of the remaining pancreas following PD was performed using CT. Five-millimeter consecutive sections were analyzed through abdominal dual phase (portal and equilibrium) helical CT. The maximum PO follow-up of the patients was 20 months. During this period all the scans performed were analyzed and also compared with the preoperative CT according to availability of data. In order to stratify the data, we considered three different PO intervals: 1–5 months of PO, 6–10 months and 11–20 months which was available in 51, 40 and 25 patients, respectively.

The analysis was performed by the operating specialist (C.S.) using the hospital’s Picture Archiving and Communication System software (PACS). As in previous studies^9,16,34,35^, the estimated level of atrophy depended on the length and thickness of the parenchyma, but the diameter of the main pancreatic duct was also measured. The length of the pancreas was taken from the extremity of the caudal portion of the pancreas (pancreatic tail) to the ostium of the celiac trunk from the aorta in the cranial portion. The thickness of the pancreas (including the main pancreatic duct) and the diameter of the main pancreatic duct (measured perpendicular to the axis of the pancreas) were measured at three different points: at 1 cm from the pancreatic tail, at 1 cm from the cranial extremity and in the middle of the pancreatic body, obtaining the same length on two sides of the measurement. The main pancreatic duct (MPD) was considered dilated when the mean diameter was higher than 3 mm (>3 mm). The volume of the gland was also calculated considering a cylindrical shape $(Volume=\pi \cdot {(\frac{T}{2})}^{2}\cdot L)$, with *T* being thickness and *L* being length.

To avoid bias in the measurement, a simple blind method was used in which the CT images were evaluated individually without providing any patient information.

### Main outcomes and confounders

The primary outcome was the development of POPF, defined and graded as in the International Study Group of Pancreatic Fistula (ISGPF)2. In this regard, grade B and C were considered clinically relevant-POPF.

Patient demographics, ASA category, consistency of the pancreas (fibrotic or soft), type of anastomosis (telescoped pancreatogastrostomy [PG], pancreaticojejunostomy [PJ] or without anastomosis [WA]), surgical technique (standard or extended PD (PPPD)), duration of the intervention, type of tumour, follow-up information (mortality or complications), administration of pancreatic enzymes, preoperative or new onset postoperative diabetes and neoadjuvant or adjuvant chemo/radiotherapy were also entered prospectively in the computer database. PJ was the technique of pancreas resection performed preferably, while PG was performed in those cases with soft pancreas and MPD < 3 mm. WA was performed in those special cases in which signs of atrophy where observed at the time of the pancreas surgery. But always taking into account the opinion of the main surgeon.

### Statistical analysis

The analysis was performed on SPSS Statistic software (SPSS, Chicago, IL, USA) and the main outcomes were compared between groups. Data were expressed as mean ± SD and we considered a value of p < 0.05 to be statistically significant. The Kolmogorov-Smirnov test was used to determine whether values followed a normal distribution and the Levene test to evaluate equality. Mean values of qualitative data were compared through the χ^2^ while quantitative data were compared with the t-student test.

In order to evaluate the morphological changes in the remnant pancreas after PD, we considered one analysis based on scans (n = 612) and a second based on patients (n = 51). In both analyses four main variables: thickness, length, diameter of the main pancreatic duct and volume of the pancreas were considered.

The first comparative scan-based study aimed to evaluate the morphological changes of the pancreas over the PO. For this, group differences in thickness, length, the diameter of the MPD and the volume were compared for the different PO intervals. Linear regression models of morphometrical changes were constructed taking into account the above-mentioned confounders. The goodness of fit of the model was assessed by the Pearson correlation coefficient (r^2^).

The second comparative study, based on the number of patients evaluated the influence of the clinical parameters studied on the four morphological variables (thickness, length, diameter of the MPD and volume). We also performed a multivariate analysis using a general linear model in which age, POPF, type of anastomosis, surgical technique, PO follow-up (time), preoperative diabetes and chemotherapy were considered as confounders.

## Data Availability

The datasets generated during and/or analysed during the current study are available from the corresponding author on reasonable request.
